# Glycosylation of Conotoxins

**DOI:** 10.3390/md11030623

**Published:** 2013-03-01

**Authors:** Gerrit J. Gerwig, Henry G. Hocking, Reto Stöcklin, Johannis P. Kamerling, Rolf Boelens

**Affiliations:** 1 NMR Spectroscopy, Bijvoet Center for Biomolecular Research, Utrecht University, Padualaan 8, 3584 CH Utrecht, The Netherlands; E-Mails: g.gerwig@rug.nl (G.J.G.); h.hocking@tum.de (H.G.H.); j.p.kamerling@uu.nl (J.P.K.); 2 Atheris Laboratories, Case postale 314, CH-1233 Bernex-Geneva, Switzerland; E-Mail: reto.stocklin@atheris.ch

**Keywords:** cone snails, glycopeptide, glycosylation, L-galactose, neuropeptide, *O*-glycan, post-translational modification, venom

## Abstract

Conotoxins are small peptides present in the venom of cone snails. The snail uses this venom to paralyze and capture prey. The constituent conopeptides display a high level of chemical diversity and are of particular interest for scientists as tools employed in neurological studies and for drug development, because they target with exquisite specificity membrane receptors, transporters, and various ion channels in the nervous system. However, these peptides are known to contain a high frequency and variability of post-translational modifications—including sometimes *O*-glycosylation—which are of importance for biological activity. The potential application of specific conotoxins as neuropharmalogical agents and chemical probes requires a full characterization of the relevant peptides, including the structure of the carbohydrate part. In this review, the currently existing knowledge of *O*-glycosylation of conotoxins is described.

## 1. Introduction

**Figure 1:**
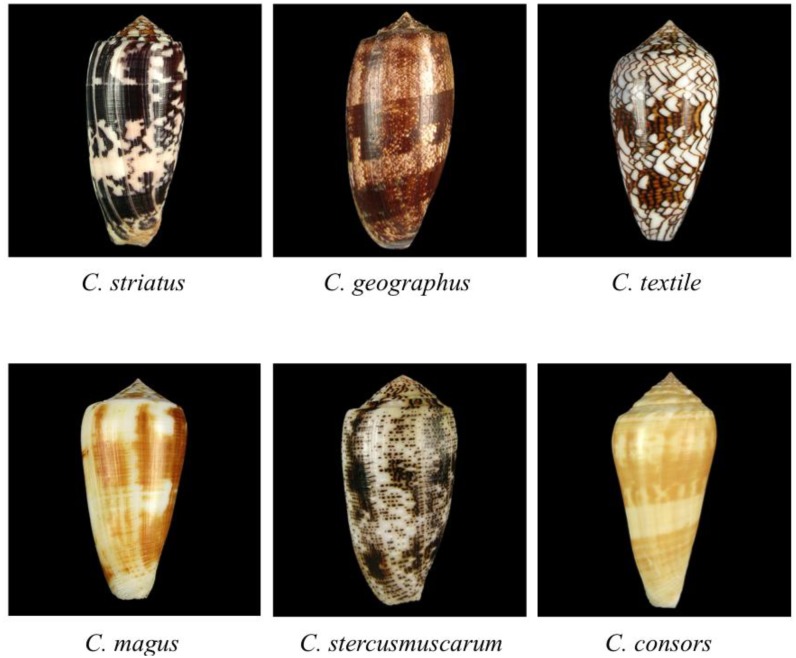
Shells of *Conus* snail species, which are discussed in this review. (Photographs ©2012 Guido and Philippe Poppe [1]).

**Figure 2:**
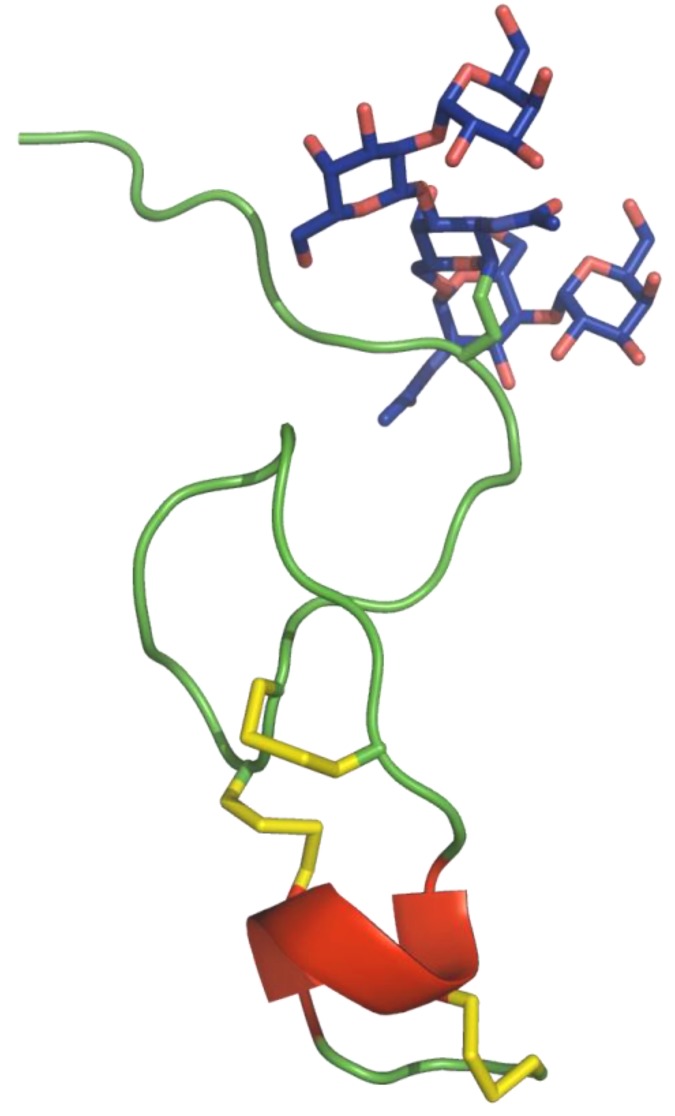
3D structure of CcTx (PDB: 4B1Q).The *O*-linked carbohydrate moieties shown in blue and disulfide bonds in yellow.

The marine predatory cone snails (genus *Conus*, family Conidae) comprise a large group of *circa* 800 different species, which are found predominantly in the West Atlantic, Caribbean, and Indo-Pacific tropical season or near coral reefs. They are a remarkable species, not only for the beauty of their conical, colorful shells (Figure 1 and [1,2]) but, in particular, for the neurotoxic compounds they use in the killing of their prey and defending themselves from predators. These compounds, called conotoxins or conopeptides, are synthesized in the venom gland of the snails [3,4]. It has been estimated that approximately 100,000 different peptides can potentially be expressed in the venoms of the entire current *Conus* genus [5,6,7,8]. However, recent studies have shown that venoms from individual cone snails revealed an unprecedented level of conopeptide diversity, expanding the predicted numbers to well in excess of 1000 unique peptides per *Conus* species [9,10,11,12]. It has to be noted that cone snail venoms also contain high molecular mass (glycosylated) proteins that participate in the envenomation cocktail [13,14], but the discussion of these compounds is out of the scope of this review.

When attacking their prey, most of the fish-hunting cone snails (e.g., *C. striatus*, *C. magus*, *C. stercusmuscarum* and *C. consors*) inject deadly venom by means of a disposable harpoon-like radular tooth into the fish (~3–50 μL at a velocity of ~200 m/s) and immobilize the fish within 2 to 3 s, before engulfing it with a large distensible rostrum [15]. In contrast, *C. geographus* uses a “net strategy” to capture the prey and to sting the fish after engulfing it first. Occasionally, humans have been injured or even killed by the sting from a cone snail, mostly *C. geographus* [16,17,18].

For many years, it has been claimed that *Conus* peptides could have great promising therapeutic applications as novel pharmacological substances in neuroscience, because they can specifically target with high affinity and specificity neurotransmitter-gated receptors and voltage-gated or ligand-gated ion channels [19,20,21,22,23,24,25].Typical examples for treatment are neurological disorders such as ischemia, spinal cord injury, Alzheimer’s disease, Parkinson’s disease, multiple sclerosis, chronic pain, stroke, epilepsy, and schizophrenia. Currently, several conotoxins are undergoing human clinical trials as therapeutic drugs [26,27,28,29,30]. One of them, ω-conotoxin MVIIA from *C. magus* venom (an *N*-type Ca^2+^ channel antagonist) is, as a synthetic product, already on the market (SNX-111/Ziconotide/Prialt™) for intrathecal treatment of severe chronic pain [31,32,33]; it is 10,000-fold more powerful than morphine. More than a billion people worldwide suffer from neuropathic pain syndromes, such as chronic pain frequently resulting from cancer, AIDS, arthritis, or injuries [34,35].

Most of the neurobiological active conopeptides are composed of 10 to 35 amino acid residues (molecular mass <5 kDa). The conopeptides contain multiple disulfide bonds and are decorated with a high variety of post-translational modifications (which can occur for up to 75% of the amino acids of a single conotoxin), leading to an exceptional diversity in peptide structures. The types of post-translational modifications found so far comprise: disulfide-bridge formation; hydroxylation of proline at C-4, lysine at C-5 and valine at γ-position; γ-carboxylation of glutamic acid (vitamin K-dependent); bromination of tryptophan at C-6; phosphorylation and sulfation of tyrosine; epimerization of L- to D-amino acids, including tryptophan, leucine, phenylalanine and valine; *C*-terminal amidation; *N*-terminal pyroglutamylation (cyclization); and *O*-glycosylation of serine or threonine.

Several comprehensive reviews have been published on the biology/biochemistry of conotoxins [36,37,38,39,40,41,42,43,44,45,46]. The review by Buczek *et al.* (2005) [42] included *O*-glycosylation data available up until 2005. Here, we will review in detail the *O*-glycosylation data of conopeptides as known in 2012.

## 2. The ConoServer Database

A specialized database for conotoxins, called ConoServer, is available online [8,47,48]. The ConoServer provides up-to-date information on the sixteen known conopeptide gene superfamilies and currently contains data for over 3500 conopeptide sequences. The ConoMass tool matches peptide masses predicted from transcripts with a list of masses obtained experimentally by proteomics analysis of cone snail venoms. Several post-translational modifications can be selected. However, it should be noted that glycosylation is not included in ConoMass because of the enormous number of possible structures glycosylation can present. Although the *O*-glycosylated conopeptides discussed in this review are included in the ConoServer, glycan information is available only for contulakin-G.

## 3. General Structural Data of Mucin-Type Glycoconjugate *O*-Glycans

*O*-Glycosylation is a common type of post(co)-translational modification of proteins in nature, resulting in the attachment of carbohydrates to hydroxyl groups of certain hydroxyl amino acid residues in the peptide backbone. Much is known about the genes and enzymes responsible for the biosynthesis of these kinds of glycoconjugates in mammalian cells [49]. The types of glycans discussed in this review belong to the so-called mucin-type glycoconjugate *O*-glycans. They are characterized by the occurrence of a carbohydrate-amino acid bond between *N*-acetyl-α-D-galactosamine and the hydroxyl function of L-serine or L-threonine. In general, the carbohydrate chains are built up from the *N*-acetyl-hexosamines (HexNAc) *N*-acetyl-D-galactosamine (GalNAc) and *N*-acetyl-D-glucosamine (GlcNAc), the hexose (Hex) D-galactose (Gal), the 6-deoxyhexose L-fucose (Fuc), and members of the sialic acid family (mainly *N*-acetyl-neuraminic acid and *N*-glycolyl-neuraminic acid). In addition, inorganic sulfate has been found as a substituent of *N*-acetyl-D-glucosamine and D-galactose. In the mucin-type *O*-glycans, three structural domains can be distinguished: the core structure, the backbone structure, and the peripheral structure. So far, besides α-D-Gal*p*NAc-(1→O) (core-type 0), nine different core types have been described (Table 1) [49,50,51,52].

**Table 1:** Known core structures of mucin-type *O*-linked glycans.

α-D-Gal*p*NAc-(1→O)	core 0
β-D-Gal*p*-(1→3)-α-D-Gal*p*NAc-(1→O)	core 1
β-D-Glc*p*NAc-(1→6)-[β-D-Gal*p*-(1→3)-]α-D-Gal*p*NAc-(1→O)	core 2
β-D-Glc*p*NAc-(1→3)-α-D-Gal*p*NAc-(1→O)	core 3
β-D-Glc*p*NAc-(1→6)-[β-D-Glc*p*NAc-(1→3)-]α-D-Gal*p*NAc-(1→O)	core 4
α-D-Gal*p*NAc-(1→3)-α-D-Gal*p*NAc-(1→O)	core 5
β-D-Glc*p*NAc-(1→6)-α-D-Gal*p*NAc-(1→O)	core 6
α-D-Gal*p*NAc-(1→6)-α-D-Gal*p*NAc-(1→O)	core 7
α-D-Gal*p*-(1→3)-α-D-Gal*p*NAc-(1→O)	core 8
α-D-Glc*p*NAc-(1→6)-[β-D-Gal*p*-(1→3)-]α-D-Gal*p*NAc-(1→O)	core 9

## 4. Glycosylated Conotoxins

### 4.1. *Conus striatus*

*C. striatus* is a fish-hunting Indo-Pacific cone snail. The mature neurotoxic conopeptide κA-conotoxin SIVA (κA-SIVA, also called s4a) isolated from its venom has been shown to be active on tetrodotoxin-sensitive voltage-gated sodium (Na_v_) channels [53]—though not on voltage-gated potassium channels as thought earlier [54]—thus eliciting spastic paralytic symptoms when injected into the fish during prey capture. The conotoxin induces intense repetitive firing of the frog neuromuscular junction leading to a tetanic contracture in muscle fiber [53]. It has a backbone of 30 amino acids with pyroglutamic acid at the *N*-terminal site, three 4-*trans*-hydroxyprolines, amidated cysteine at the *C*-terminal side, and three disulfide bonds. The peptide contains three serine residues, with a glycan at one of them, and three threonine residues. The primary structure of the glycopeptide is included in Table 2.

Using electrospray-mass spectrometry (ESI-MS), a Hex_3_HexNAc_2_ glycan moiety (892.8 Da) was identified at Ser-7 [54]. This was the first evidence for *O*-glycosylation as a post-translational modification in a biological active conopeptide. The MS/MS spectrum of the peptide revealed HexNAc_2_, HexHexNAc_2_, Hex_2_HexNAc_2_ and Hex_3_HexNAc_2_ fragment ions, and showed losses of one, two and three Hex residues from the intact pseudomolecular ion. The nature of the monosaccharides and type of linkages were not determined. Later, the Hex_3_HexNAc_2_ glycan moiety (893 Da) of κA-SIVA (s4a) was confirmed by Jakubowski *et al.* [55] using LC/ESI-MS, and by Kelley *et al.* [53] using MALDI-TOF-MS and LC/ESI-MS. Evidence was presented for the occurrence of a HexNAc-HexNAc fragment, to which three Hex residues are connected. MS analysis of the ammonia-treated material (β-elimination reaction removing the *O*-glycan) supported the MS results of the native material, but there was no information about the nature and linkage types of the sugar units.

**Table 2:** Overview of *O*-glycosylated conotoxins.

*Conus* species	Diet	Conotoxin name(s)	Glycopeptide sequence	*O*-linkedresidue	*O*-glycan	References
*C. striatus*	P	κA-SIVA	ZKSLVPS*VITT*CC*GYDOGTM*C*OO*C*RCTNS*C*-NH_2_	Ser-7	Hex_3_HexNAc_2_	[53,54,55,56,57]
		(s4a)				
*C. striatus*	P	κA-SIVB	ZKELVPS*VITT*CC*GYDOGTM*C*OO*C*RCTNS*C*OTKOKKO-NH_2_	Ser-7	Hex_3_HexNAc_2_	[53,56,57]
		(s4b)				
*C. stercusmuscarum*	P	SmIVA	ZTWLVPS*T*ITT*CC*GYDOGTM*C*OT*C*M*C*DNT*C*KOKOKKS-NH_2_	Ser-7	No information	[57]
		(κA-SmIVA)		Thr-8	No information	
*C. stercusmuscarum*	P	SmIVB	AOWLVPS*T*ITT*CC*GYDOGSM*C*OO*C*M*C*NNT*C*KOKOKKS-NH_2_	Ser-7	No information	[57]
		(κA-SmIVB)		Thr-8	No information	
*C. consors*	P	CcTx	AOWLVPS*QITT*CC*GYNOGTM*C*OS*C*M*C*TNT*C*	Ser-7	Hex_2_HexNAc_2_	[10,11,15,52,58]
		(κA-CcTx)			Gal_3_GlcNAcGalNAc	
*C. magus*	P	κA-MIVA	AOγLVVT*AT*TN*CC*GYNOMTI*C*OO*C*M*C*TYS*C*OOKRKO-NH_2_	Thr-7	Hex_4_HexNAc_2_ as sum of both sites	[57]
				Thr-9		
*C. geographus*	P	contulakin-G	ZSEEGGSNAT*KKPYIL	Thr-10	β-D-Gal*p*-(1→3)-α-D-Gal*p* NAc	[36,59,60,61,62,63]
					SO_4_(HexHexNAc) Hex_3_	
		(CGX-1160)			Hex_2_HexNAc_2_	
*C. textile*	M	ε-TxIX	γ*CC*γDGW*CCT*AAO	Thr-10	α-D-Gal*p*-(1→3)-α-D-Gal*p*NAc	[41,64,65,66]
		(tx5a, TxVa or Tx-012)				

P = piscivorous; M = molluscivorous; Z = pyroglutamic acid; S* = glycosylated serine; T* = glycosylated threonine; O = 4-*trans*-hydroxyproline; γ = γ-carboxyglutamic acid; W* = 6-bromotryptophan.

Preliminary results with the synthetic non-glycosylated κA-conotoxin analog indicated that this was far less potent when injected into animals than the native glycosylated κA-conotoxin [54]. Suggested plausible roles for the *O*-glycosylation included increasing the on-time and/or affinity of the peptide for its ion channel and increasing the speed of access of the peptide to the channels. See also a mini-review by Craig *et al.* [36].

The homologous conotoxin, κA-conotoxin SIVB (κA-SIVB, also termed s4b) is built up from 37 amino acids with *N*-terminal pyroglutamic acid, *C*-terminal amidated 4-hydroxyproline, and three disulfide bridges [53]. The primary structure of the conopeptide is included in Table 2.

κA-SIVB, in combination with κA-SIVA, is a major component of the injected venom of *C. striatus* [56] and has a similar neuroexcitatory profile as κA-SIVA. In fact, both conotoxins mimic the biological effects of the completely injected venom on fish prey. κA-SIVB was also reported to be glycosylated at Ser-7 [53,56,57]. Based on MALDI-TOF-MS and LC/ESI-MS combined with ammonia treatment, the occurrence of Hex_3_HexNAc_2_ was suggested [53], a similar type of glycosylation as found for κA-SIVA (s4a).

### 4.2. *Conus geographus*

The major peptide in the venom of the fish-hunting *C. geographus*, which lives in the Philippine seas, is a 16-amino acid glycopeptide called contulakin-G [59]. This conotoxin, a neurotensin subtype 1 (NTS1) receptor agonist, targets G-protein-coupled receptors. It was shown to be a potent analgesic when administered intrathecally in animal models [67,68]. Peptide analysis studies showed the presence of a modified Thr-10 residue. A further post-translational modification is a pyroglutamic acid residue at the *N*-terminal site. The primary structure of the glycopeptide is included in Table 2.

**Scheme 1:**
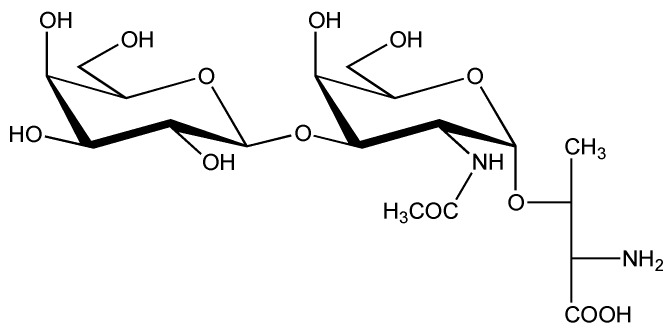
Structure of β-D-Gal*p*-(1→3)-α-D-Gal*p*NAc-(1→O)-L-Thr.

A combination of MALDI-TOF-MS, LSI-MS, and ESI-MS determined that the major *O*-glycoform corresponded with the Hex-HexNAc sequence. Additionally, three less abundant glycosylated forms were observed, *i.e.*, SO_4_(HexHexNAc), Hex_3_, and Hex_2_HexNAc_2_ [59]. Enzymatic experiments (a β-D-galactosidase preferentially hydrolyzing (β1→3)-linked D-galactopyranosyl residues and an *O*-glycosidase treatment liberating a disaccharide) and MALDI-TOF-MS identified the major *O*-glycoform as a core-1 type structure, β-D-Gal*p*-(1→3)-α-D-Gal*p*NAc-(1→O)-, as depicted in Scheme 1. This is the T-antigen, one of the most common eukaryotic *O*-glycan structures [49]. The native contulakin-G coeluted on RP-HPLC with synthetic contulakin-G containing the same disaccharide [59].

When administered to mice, the synthetic glycopeptide produced similar neurological effects as found for the native material (motor control-associated dysfunction). However, the glycosylated form was active at 10-fold lower doses than the non-glycosylated form. In contrast, comparing the binding activities of the synthetic glycopeptide and the synthetic peptide for a number of neurotensin receptor types yielded weaker affinities for the glycosylated material. The different results between the *in vivo* and *in vitro* studies, when focused on the importance of the *O*-glycosylation, are contradictory. Additional studies showed that the proteolytic degradation of contulakin-G is inhibited by the presence of the *O*-glycan, which may lead to an enhanced supply *in vivo* of the glycopeptides to the receptor. However, alternative explanations were not excluded and should be studied in more detail [36,42,59]. The glycosylated form is, in fact, a very potent broad-spectrum analgesic, being two orders of magnitude more potent than the non-glycosylated form *in vivo* [19,69]. It should be noted that contulakin-G has entered phase II clinical trials for short-term management of post-operative pain.

In a few related studies, NMR spectroscopy was used to investigate the three-dimensional structure of contulakin-G and some synthetic analogs, although the analogs showed lower bioactivity than the native contulakin-G.

In the first study, NMR solution conformations were reported for native contulakin-G with β-D-Gal*p*-(1→3)-α-D-Gal*p*NAc-(1→O)- at Thr-10, its non-glycosylated variant, and two glycopeptide analogs, one containing α-D-Gal*p*NAc-(1→O)- at Thr-10 and the other containing β-D-Gal*p*-(1→3)-α-D-Gal*p*NAc-(1→O)- at Ser-7 [60]. It was found that all four substances have mainly random coil peptide conformations. Interestingly, in the glycosylated peptides, transient populations of folded conformations are present. The restricted rotation of α-D-Gal*p*NAc at Thr-10 around the linkage between the glycan and the peptide was explained by intramolecular hydrogen bonding between the amide proton of GalNAc and most likely the carbonyl oxygen of Thr-10 in the peptide chain. Such a hydrogen bond was not seen for the peptide *O*-glycosylated at Ser-7. A comparison of the activities of the four compounds in an assay of acute pain (ability to induce latency of tail flick in mice) demonstrated that a reduction of the size of the glycan, or a shift in the position of the glycosylation site, decreases the activity with respect to contulakin-G itself. Therefore, it was suggested that the stabilization of the peptide conformation by hydrogen bonding to the carbohydrate could be a key factor in the biological activity [60]. In this context, it should be noted that the T-antigen at Thr-10 showed significant protection against enzymatic degradation by Pro-specific endopeptidase, but when attached to Ser-7, this protection was completely abolished. Based on these data, it was hypothesized that it is the orientation of the glycan chain relative to the peptide chain that is actually recognized by the proteolytic enzyme [61].

In a subsequent study, the NMR solution conformations of the [L-Ser-10] and [D-Ser-10] analogs of contulakin-G were reported [61], and subtle differences in conformational preferences between the analogs and native contulakin-G were found. In fact, the intramolecular hydrogen bonding as occurring in native contulakin-G was lacking. Interestingly, the biological activity of the [D-Ser-10] analog of contulakin-G was similar to that of contulakin-G itself. Thus the hydrogen bond between the glycan and the peptide in contulakin-G seems not to direct the biological activity. The [L-Ser-10] analog showed some activity at more than 100 times the dose.

**Scheme 2:**
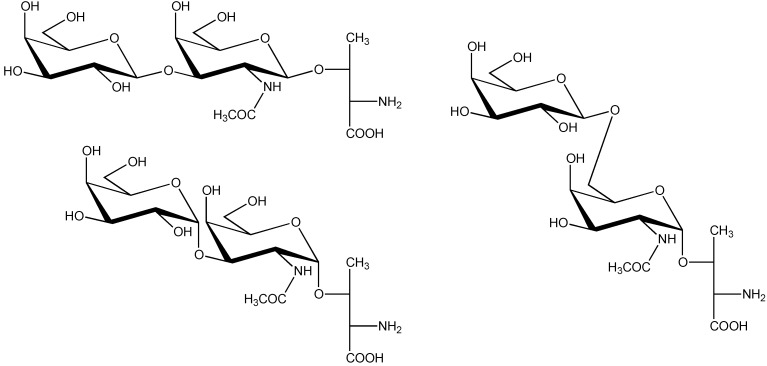
Structures of β-D-Gal*p*-(1→3)-β-D-Gal*p*NAc-(1→O)-L-Thr, α-D-Gal*p*-(1→3)-α-D-Gal*p*NAc-(1→O)-L-Thr and β-D-Gal*p*-(1→6)-α-D-Gal*p*NAc-(1→O)-L-Thr.

In another study, for direct comparison with contulakin-G comprising β-D-Gal*p*-(1→3)-α-D-Gal*p*NAc-(1→O)- at Thr-10, three analogs with different *O*-glycans at Thr-10, *i.e.*, β-D-Gal*p*-(1→3)-β-D-Gal*p*NAc-(1→O)-, α-D-Gal*p*-(1→3)-α-D-Gal*p*NAc-(1→O)-, and β-D-Gal*p*-(1→6)-α-D-Gal*p*NAc-(1→O)-, respectively, were synthesized [62] (see Scheme 2), but so far biological and conformational details are missing.

A study focusing on the enzymatic glycosylation of the non-glycosylated form of contulakin-G showed that the mammalian UDP-D-GalNAc:polypeptide:α-GalNAc-transferase T1 (ppGalNAc-transferase T1, EC 2.4.1.41) was able to transfer GalNAc from UDP-GalNAc to Thr-10 of the peptide backbone, although Ser-7 was also glycosylated to some extent [63]. It is not clear if this glycosylated product was tested for bioactivity.

### 4.3. *Conus textile*

The glycosylated conotoxin ε-TxIX (also called tx5a, Tx-012 or TxVa), with a backbone of 13 amino acids, occurs as the most abundant peptide in the venom of the mollusc-hunting cone snail *C. textile*. Nine out of the thirteen amino acids are post-translationally modified [64]. The post-translational modifications comprise: γ-carboxyglutamic acid, 6-bromotryptophan, 4-*trans*-hydroxyproline at the *C*-terminus, and *O*-glycosylation at Thr-10, in addition to two disulfide bridges (Table 2). When injected intracerebroventricularly into mice, it causes hyperactivity and spasticity. It is suggested that the glycopeptide may target presynaptic calcium channels (blocker) or act on G protein-coupled presynaptic receptors via another mechanism [64]. Using monosaccharide analysis and MALDI-TOF-MS, the *O*-glycan was defined as a disaccharide Gal-GalNAc. Despite no reference to an analysis of the linkage types and anomericities of the carbohydrate constituents, the solution structure of ε-TxIX was determined by NMR spectroscopy and showed a high flexibility of the disaccharide moiety [64].

In a parallel report, the same structure (as Hex-HexNAc) was presented, as determined by MALDI-TOF-MS and ESI-MS/MS [65]. The Hex and HexNAc residues were identified to be galactose and *N*-acetylgalactosamine, but no linkage type was reported.

Detailed structural information on ε-TxIX, obtained by 1D and 2D NMR (COSY, HSQC, NOESY) spectroscopy [66], yielded a core-8 type structure, α-D-Gal*p*-(1→3)-α-D-Gal*p*NAc-(1→O)- (see Scheme 2). Note that the anomeric configuration of the terminal galactose residue (α) is the only difference with the *O*-glycan structure (T-antigen; see Scheme 1) determined for conopeptide contulakin-G from *Conus geographus* venom. The inability to split off terminal galactose using β-galactosidase inferred the presence of terminal α-galactose in tx5a. Likewise, the inability to split off the disaccharide moiety with endo-*O*-glycosidase (endo-α-*N*-acetylgalactosaminidase) inferred interglycosidic linkages distinct from those found in the T-antigen [66]. However, the absolute configuration (D) of the monosaccharide constituents was not actually determined.

### 4.4. *Conus magus*

κA-conotoxin MIVA (κA-MIVA), the 36-amino acid peptide from the venom of the fish-hunting species *C. magus*, causes the same spastic symptomatology as κA-SIVA [57]. The conopeptide contains three disulfide bonds and has ten post-translationally modified amino acids. These include seven hydroxylated proline residues, including one *C*-terminal, a γ-carboxy-glutaminic acid and two modified threonine residues (may be *O*-glycosylated), *i.e.*, Thr-7 and Thr-9 [57]. The amino acid sequence of the conopeptide is depicted in Table 2. *O*-Glycan details were not included for κA-MIVA, but we noted that the mass difference (1053.6 Da) found in LSI-MS studies of the native glycopeptide as compared to the unglycosylated form agrees with a composition Hex_4_HexNAc_2_ shared between the two glycosylation sites.

### 4.5. *Conus stercusmuscarum*

The 37-amino acid κA-conotoxins SmIVA and SmIVB from the venom of the Indo-Pacific fish-hunting species *C. stercusmuscarum* elicit a spastic paralysis upon injection of venom into the fish during prey capture [57]. Both compounds were suggested to be *O*-glycosylated at Ser-7 and Thr-8, but details about the glycans present have not been published. The predicted mature toxin primary structure is included in Table 2.

### 4.6. *Conus consors*

The conotoxin CcTx, isolated from the venom of the Indo-Pacific fish-hunting cone snail *C. consors* (Pionoconus clade) produced a marked contraction and extension of the caudal and dorsal fins upon injection into fish [58]. When tested on isolated frog neuromuscular preparations, CcTx showed skeletal muscle contractions, indicating a potent excitotoxin that targets tetrodotoxin-sensitive voltage-gated sodium channels. It selectively increases motor nerve terminal excitability resulting in repetitive and spontaneous action potential that lead to sudden titanic paralysis of the prey. The conopeptide belongs to the κA-family of conotoxins, having 73% sequence homology with κA-SIVA (from *C. striatus* venom) and the same cysteine scaffold [58].

Chemical microsequencing and ESI-MS revealed a peptide of 30 amino acids with three disulfide bridges, a *C*-terminal cysteine residue and three 4-hydroxyproline residues [15,58]. A post-translational modification involving an *O*-glycosylation of a Ser or Thr residue at position 7 was suggested. Additional studies on the composition of dissected venom versus milked venom of *C. consors* yielded CcTx as a major compound in both sources. Using RP-HPLC followed by MALDI-TOF-MS and ESI-MS, the *O*-glycosylation site was fixed at Ser-7, although the composition of the *O*-glycan was not determined yet (Table 2) [10]. Besides CcTx (MW = 4118.2 Da), a second major compound of a higher molecular mass (5179.7 Da) was detected in the venom, specified as an unknown glycosylated peptide (“CcTx-like”) [11]. Furthermore, a partially deglycosylated CcTx component (3953.7 Da), missing one Hex residue (162 Da), was observed. The latter long-term study demonstrated that the injected venoms of *C. consors* individuals are not constant in peptide composition and can drastically vary with time.

Recently, a detailed investigation of the *O*-glycan of CcTx has been reported [52]. Using MALDI-TOF-MS and ESI-MS the carbohydrate chain at Ser-7 could be described as Hex_3_HexNAc_2_. Using monosaccharide analysis, absolute configuration determination, methylation analysis and NMR spectroscopy, the complete structure for the *O*-glycan chain of CcTx was determined to be α-L-Gal*p*-(1→4)-α-D-Glc*p*NAc-(1→6)-[α-L-Gal*p*-(1→2)-β-D-Gal*p*-(1→3)-]α-D-Gal*p*NAc-(1→O)-.

**Scheme 3:**
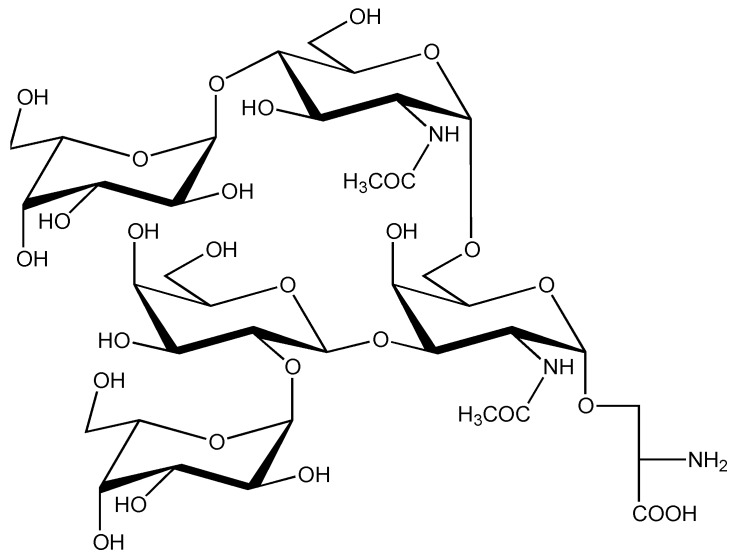
Structure of α-L-Gal*p*-(1→4)-α-D-Glc*p*NAc-(1→6)-[α-L-Gal*p*-(1→2)-β-D-Gal*p*-(1→3)-]α-D-Gal*p*NAc-(1→O)-L-Ser.

This *O*-glycan (see Scheme 3) has completely novel structural features. Besides a conventional β-D-Gal*p*-(1→3)-α-D-Gal*p*NAc-(1→O)- fragment, which also occurs in contulakin-G and in many mucin-type glycosylations, the α-D-Gal*p*NAc- unit is substituted at O6 with an α-D-Glc*p*NAc-(1→6)- unit, yielding a novel core-type structure α-D-Glc*p*NAc-(1→6)-[β-D-Gal*p*-(1→3)-]α-D-Gal*p*NAc-(1→O)-, which was defined as core-type 9. However, the most remarkable finding was the occurrence of terminal α-Gal*p*- residues at the upper and lower branch, both having an L-configuration, which makes the *O*-glycan even more unique. Analysis of the NMR solution structure of CcTx (Figure 2) showed that the backbone of the *C*-terminal region was well defined with three disulfide bridges, a series of turns, including a Type I’ β-turn for Cys-12–Tyr-15 and a partially distorted Type I β-turn for Asn-16–Thr-19, preceding the short α-helix Ser-23–Thr-27.

The *N*-terminal region Ala-1–Thr-11 appears less well defined, but presents a kink in the backbone centered on the *O*-glycosylated Ser-7. Although the *N*-terminal region is more disordered, the pentasaccharide orients itself consistently on one side of the peptide, with the α-D-Gal*p*NAc and, to a lesser extent, the α-D-Glc*p*NAc residue oriented towards the peptide chain [52].

## 5. Peptide Sequence Comparison

Unlike the Asn-Xxx-Ser/Thr consensus amino acid sequence for *N*-glycosylation, no consensus target sequence has been identified for *O*-glycosylation of proteins/peptides [70], let alone that of conotoxins. However, an interesting pattern has emerged from the comparison of the peptide sequences for members of the κA-conotoxin family [52]. These sequences, known and predicted from cDNA, can be divided into three groups, each distinct from one other in terms of level of glycosylation. The first group comprises the κA-conopeptides that have an *O*-glycosylated serine residue at position 7 in their respective sequences. The conotoxins CcTx (*C. consors*), κA-SIVA (*C. striatus*), κA-SIVB (*C. striatus*), SmIVA (*C. stercusmuscarum*), and SmIVB (*C. stercusmuscarum*) belong to this group. As shown in Table 2, there is a significant peptide sequence identity between these five compounds. The closest homolog to CcTx is κA-SIVA, showing 73% sequence identity. As previously mentioned, CcTx, κA-SIVA, and κA-SIVB have been shown to contain an *O*-glycan with the composition Hex_3_HexNAc_2_ and for CcTx the carbohydrate structure has been elucidated in detail. We suggest that the *O*-glycans of κA-SIVA and κA-SIVB could have the same unusual primary structure as found for CcTx. The second group comprises the κA-conopeptides that are predicted to have two glycosylated threonine residues at position 7 and 9, as demonstrated for κA-MIVA (*C. magus*). So far, structural details of the *O*-glycan are missing. The third group, known as short κA-conotoxins, lacks the *O*-glycosylated *N*-terminal tail present in the other two groups.

Contulakin-G (*C. geographus*) and ε-TxIX (*C. textile*) are the smallest conotoxins and contain no sequence similarity to other conopeptides as shown in Table 2, except that they both have a glycosylated threonine residue at position 10. However, their *O*-glycan disaccharides differ in the anomeric configuration (β *versus* α, respectively) of the non-reducing D-Gal*p* residue (*vide supra* and Table 2).

## 6. Biosynthesis and Roles of *O*-Glycosylation in Conotoxins

Several recent reviews are available in the literature describing the current knowledge of the peptide biosynthesis of conotoxins [42,71,72,73,74]. As mentioned already in the Introduction, many constituent amino acids of the peptides are post-translationally modified. The biosynthesis of the many different conotoxins is probably associated with the specific type of epithelial cells found in different sections of the venom duct of the snail. For instance, qualitative and quantitative differences in conotoxin components were found in the proximal, central and distal sections of the *C. textile* and *C. geographus* venom duct, suggesting specialization of duct sections for the biosynthesis of particular conotoxins [75,76,77]. However, most biochemical and cellular events that occur in the venom duct have not yet been fully characterized.

Although the pathways of *O*-glycosylation for conotoxins have not been outlined in detail, the observation that the synthetic non-glycosylated contulakin-G peptide could be glycosylated by a mammalian UDP-D-GalNAc: polypeptide α-GalNAc-transferase (*i.e.*, ppGalNAcT1) [63]) points to a similar pathway for *O*-glycosylation in *Conus* species as in mammals. This means that all monosaccharides are added one at a time in a stepwise series of reactions, in contrast to the formation of a lipid-linked precursor oligosaccharide followed by *en bloc* transfer of the oligosaccharide to the polypeptide in *N*-glycosylation. The *O*-glycosylation starts with the transfer of D-Gal*p*NAc from UDP-α-D-Gal*p*NAc to a Ser or Thr residue of the peptide backbone. The attachment of α-D-Gal*p*NAc is catalyzed by one of the members of the large ppGalNAcT family, yielding the α-D-Gal*p*NAc-(1→O)-Ser/Thr element [49,50]. As mentioned before, there are no simple peptide target sequences for *O*-glycosylation, analogous to the Asn-Xxx-Ser/Thr sequences for *N*-glycosylation, and it has to be noted that ppGalNAcTs differ in their specificity for different sequences of amino acids surrounding the glycosylation target. However, a preponderance of adjacent Pro and Ala residues has been associated with sites of *O*-glycosylation [70]. Pro residues appear to influence protein conformation by breaking helix formation and promoting the formation of β-turns and β-sheets. In subsequent reactions, additional monosaccharides are transferred individually from nucleotide sugar donors to the growing *O*-glycan chain by a variety of glycosyltransferases. The core-1 type disaccharide β-D-Gal*p*-(1→3)-α-D-Gal*p*NAc, as found in contulakin-G (*C. geographus*), is the major core type found in mammals, and its biosynthesis using mammalian β-1,3-D-galactosyltransferase (core 1 β3GalT) has been well described [50]. A similar transferase is expected to be present in *C. geographus*. For the core-8 type disaccharide α-D-Gal*p*-(1→3)-α-D-Gal*p*NAc, present in ε-TxIX (*C. textile*), and found earlier in human bronchial tissues [78], no gene potentially encoding this α-1,3-D-galactosyltransferase has been identified. However, such an enzyme is well known for the biosynthesis of the Galili epitope α-D-Gal*p*-(1→3)-β-D-Gal*p*-(1→4)-β-D-Glc*p*NAc(1→ [79,80,81]. The recently identified novel core-9 type fragment α-D-Glc*p*NAc-(1→6)-[β-D-Gal*p*-(1→3)-]α-D-Gal*p*NAc in the pentasaccharide *O*-glycan of CcTx of *C. consors* [52] would require an α-1,6-*N*-acetyl-D-glucosaminyltransferase, an enzyme that also has not yet been described. Moreover, for the biosynthesis of the complete pentasaccharide in CcTx, two other unknown glycosyltransferases are needed, namely, α-1,4-L-galactosyltransferase and α-1,2-L-galactosyltransferase, as well as the nucleotide L-Gal*p* donor.

It is important to consider the biological significance of these complex carbohydrates of conotoxins. The biological functions of glycoprotein/glycopeptide glycans can be roughly divided into two broad categories: (1) intrinsic functions performed by glycans, such as providing structural components and modifying physiological properties; (2) extrinsic functions resulting from glycan–protein or glycan–glycan interactions, such as directing trafficking, mediating and modulating cell-adhesion and signaling [82,83]. In the case of conotoxins, it has been reported that the post-translational modifications of amino acids increased the toxin potency [36,38,42,72,84] and assisted the stabilization of the three-dimensional molecular structure [60,66,85]. This fits the general agreement that the glycosylation of peptides may increase their biological stability by slowing down the proteolytic degradation of the polypeptide backbones (as a means of protection), as well as by stabilizing their tertiary structures, thereby increasing their lifespans. Nevertheless, in the whole array of biological events, carbohydrate-recognizing receptors and lysosomal catabolic enzymes might also play an important role. As mentioned earlier, it was speculated that the role of the *O*-glycan (T-antigen) in contulakin-G could be to increase the stability *in vivo*, thereby enhancing the bioavailability of the toxin at the receptor site [36,54,59,61]. With regards to lysosomal catabolism, it should be noted that relatively little is known about *O*-glycans in comparison with *N*-glycans [86,87]. *O*-Glycosylation could be responsible for enhancing the stability of CcTx (*C. consors*) *in vivo* and the presence of terminal α-L-galactose residues in the upper and lower branch of the glycan chain (Scheme 3) may further enhance this stability. Indeed, glycoconjugates generally contain D-galactose residues, which can be released with conventional D-galactosidases in catabolic pathways. This would mean that in preys devoid of L-galactosidases, the presence of terminal α-L-galactose could provide an extra level of protection against the breakdown of CcTx. However, to the best of our knowledge, the presence of α-L-galactosidases in fish, being the preys of *C. consors*, has not been reported. It is, however, known that marine microorganisms can express α-1,3-(3,6-anhydro)-L-galactosidase and α-1,3-(6-sulfate)-L-galactosidase, enzymes that catalyze the degradation of agar, the major component of the cell walls of red algae [88,89]. But the roles served by glycosylation and many other post-translational modifications in conotoxins remain, for the most part, unexplored. In higher mammals, α-L-galactosidases have not been reported, which is of interest, considering the potential applications of these conotoxins in the treatment of human neurological disorders.

It is clear that the study of the biological role of the glycan in conotoxins requires an unambiguous determination of the identity and quantity of the glycan species. Determining the 3D structure of the complete peptides, elucidated using NMR and molecular dynamics, is crucial to our understanding of the structure–activity relationship of these peptides [52,85,90,91].

## 7. Concluding Remarks

Cone snail venom will continue to attract a growing interest as a vast untapped biological resource [92,93]. Conotoxins have proven effective in drug design and could be used to treat various disorders such as schizophrenia, neuromuscular disorders, and chronic pain. Nowadays, the venoms of more than 500 species of cone snails are being systematically characterized. This is an overwhelming task because each *Conus* species contains hundreds of peptides in its venom, and, overall, these peptides exhibit high amino acid sequence diversity, both between species and within species [9,11,90,94]. Moreover, it has been observed that the venom variations between individuals of a single geographical population are greater than variations observed between geographical distant populations. The significant inter- and intra-species differences in the venom repertoire constitute an intriguing challenge for scientists investigating the proteins involved in biosynthesis, modification and secretion of such an enormous diversity of compounds (a field called “Venomics”) [95,96]. The current knowledge of the glycosylation of conopeptides has been summarized in Table 2. Thus far, it seems that glycosylation occurs mostly in fish-hunting species.

We have observed that, since the discovery of a possible glycosylation of conotoxins, little or no attention has been paid to the detailed structural analysis of the conopeptide glycans. One can only speculate about the reasons why this is the case, but perhaps this aspect has simply been overlooked. It is hoped that this review will contribute to an increase of glycobiology activities in the venomics field.
